# Continuum of care in maternal and child health in Indonesia

**DOI:** 10.1017/S1463423624000094

**Published:** 2024-04-19

**Authors:** Anu Rammohan, Srinivas Goli, Hoi Chu

**Affiliations:** 1 Department of Economics, University of Western Australia, Perth, WA, Australia; 2 Department of Fertility and Social Demography, International Institute for Population Sciences (IIPS), Mumbai, India

**Keywords:** continuum of care, maternal and child health, Indonesia

## Abstract

**Aim:**

This paper aims to empirically analyze the socioeconomic and demographic correlates of maternal and child health (MCH) care utilization in Indonesia using the *continuum of care* (CoC) concept.

**Background:**

The concept of CoC has emerged as an important guiding principle in reproductive, maternal, newborn, and child health. Indonesia’s maternal mortality rate, neonatal mortality, and under-five mortality rates are among the highest in the Southeast Asian region.

**Methods:**

Using pooled data from four successive waves of the nationally representative Indonesian Demographic and Health Survey (IDHS) conducted in the years 2002, 2007, 2012, and 2017, we use multivariate regression models to analyze care across four components of the continuum: antenatal care (ANC), institutional delivery, postnatal care for children, and full immunization (IM).

**Findings:**

CoC at each stage of MCH care has improved continuously over the period 2002–2017 in Indonesia. Despite this, just less than one out of two children receive all four components of the CoC. The overall coverage of CoC from its second stage (four or more ANC visits) to the final stage (full child IM) is driven by the dropouts at the ANC visit stage, followed by the loss of postnatal checkups and child IM. We find that the probability of a child receiving CoC at each of the four stages is significantly associated with maternal age and education, the household’s socioeconomic and demographic characteristics, and economic status.

**Conclusion:**

Complete CoC with improved, affordable, and accessible MCH care services has the potential to accelerate the progress of Sustainable Development Goal 3 by reducing maternal and childhood mortality risks. Our findings show that in Indonesia, the CoC continuously declines as women proceed from ANC to other MCH services, with a sharp decline observed after four ANC visits. Our study has identified key socioeconomic characteristics of women and children that increase their probability of failing to access care.

## Introduction

Pregnancy and childbirth are life-threatening for millions of women in low-income countries due to difficulties in accessing life-saving maternal healthcare services. Globally, one woman loses her life every 2 min during pregnancy and childbirth (WHO, UNICEF, UFPA, World Bank Group and UNDESA/Population Division, 2023). Similarly, in the critical neonatal period, an estimated 2.3 million babies died in the first month of life in 2021, and there were 5.0 million deaths among children aged under five years. Infectious diseases, such as pneumonia, diarrhea, and malaria, along with preterm birth and intrapartum-related complications are among the leading contributors to these deaths (UNICEF, 2023). A significant proportion of these deaths are preventable if there is adequate access to maternal and child health (MCH) care services. Although there has been significant global progress in reducing both child and neonatal mortality, they remain unacceptably high.

To address issues with poor MCH outcomes, over the last decade or so, the concept of *continuum of care* (CoC) has emerged as an important guiding principle in reproductive, maternal, newborn, and child health (Tinker *et al*., 2005; Kerber *et al*., 2007; Dean *et al*., 2014). The provision of adequate MCH care in the continuum is crucial for achieving Goal 3 of the United Nation’s Sustainable Development Goals (SDGs) which seeks to ensure ‘good health and well-being’ (United Nations, 2019).

The CoC concept, originally proposed by Tanahashi (1978), involves an integrated package of maternal, newborn, and child health services from pregnancy to the postnatal period. CoC is typically defined as a continuity in the care-seeking practices and behavior for maternal and newborn health. This includes improving access to antenatal care (ANC) during pregnancy, improved management of normal delivery by skilled attendants, access to neonatal care when needed, postnatal care for both mothers and newborns, and timely immunization (IM) of children below five years of age.

The CoC concept is particularly critical for newborn and maternal health in developing countries with high maternal and child mortality rates (Rammohan *et al*., 2021). The CoC concept has been used to study maternal and healthcare usage in Pakistan (Iqbal *et al*., 2017), Ghana (Yeji *et al*., 2015), and Cambodia. These studies have found that the completion of CoC was low in Ghana (8%) and Pakistan (27%). For Ghana, the greatest decline in healthcare usage was between delivery and postnatal care within 48 hours postpartum (Yeji *et al*., 2015). In Pakistan, CoC completion was found to be higher among better-educated women with better autonomy and socioeconomic backgrounds (Iqbal *et al*., 2017).

This paper aims to empirically analyze the socioeconomic and demographic correlates of MCH care utilization in Indonesia using the CoC concept. Although Indonesia has made significant progress in improving MCH outcomes, maternal mortality has been stagnant between 1994 and 2014 (Agustina *et al*., 2019); Indonesia’s maternal mortality ratio is among the highest in Southeast Asia (UNFPA, 2023). Furthermore, despite Indonesia making significant progress in reducing under-five and neonatal mortality, they remain among the highest in Southeast Asian region (UNICEF, 2023). Moreover, IM rates among children have remained largely stagnant, with a significant number of children missing out on important childhood vaccinations. By 2017, over 20% of Indonesian children remained unvaccinated or were only partly immunized (Chu and Rammohan, 2022) with rural–urban and socioeconomic differences in measles vaccination rates (Fernandez *et al*., 2011; Rammohan *et al*., 2012). Although access to MCH services has improved in Indonesia, there continue to be gaps in priority interventions such as family planning, care during childbirth, and management of childhood illness (Soedarmono, 2017). There are also large regional disparities across Indonesia in access to ANC and skilled attendants at birth (Tripathi and Singh, 2017; Laksono *et al*., 2020), with the eastern provinces having relatively poorer MCH outcomes.

Previous research has found socioeconomic and demographic factors such as education and wealth status as being important predictors for accessing care across the continuum in Indonesia (Nafiah *et al*., 2022; Andriani *et al*., 2022) and in other lower- and middle-income countries (Osaki *et al*., 2013; Yeji *et al*., 2015; Wang and Hong, 2015; Iqbal *et al*., 2017; Addisu *et al.,* 2022).

From a policy perspective, identifying the gaps in MCH services and the socioeconomic characteristics that affect access to care is critical for reducing maternal and early childhood mortality rates and in addressing regional differences in healthcare services. However, despite the large international literature on CoC, there is limited research from Indonesia using the CoC concept. Indonesia-focused studies on CoC in MCH care have largely used one survey year (Andriani *et al*., 2022) or data from home-based records in MCH care (Osaki *et al*., 2013; 2015). Others have focused on a single component of MCH such as access to ANC (Nafiah *et al*., 2022; Andriani *et al*., 2022). This would make it difficult to identify the point at which health-seeking behavior falls and the regional variations in access to care.

**Figure 1.**
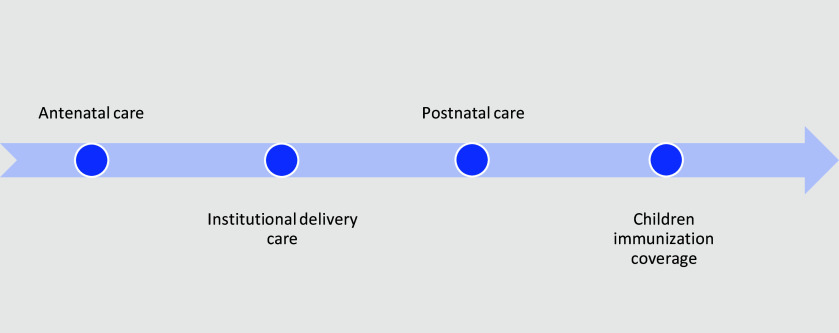
Framework of continuum of care in maternal and child health service utilization.

Given this background, our study provides a more nuanced and policy-focused analysis using the CoC framework in MCH care by including care from antenatal to childhood IM completion. Specifically, we analyze care across four components of the continuum: ANC, institutional delivery (ID), postnatal care for children (PNC), and full IM (Figure 1).

Our paper addresses a significant gap in the literature by charting the discontinuities in CoC to identify the socioeconomic and demographic factors influencing women at risk of falling behind in access to maternal and childcare.

The paper makes four contributions to the literature: first, the data for our analysis come from four cross-sectional waves of the nationally representative Indonesian Demographic and Health Survey (IDHS). This allows us to analyze CoC in MCH care from the first ANC visit to child IM. This fills a significant research gap as previous Indonesian research focused on CoC up to the postnatal care stage using a single survey year. Using data over a longer period allows us to examine trends in CoC in MCH over time, identify the socioeconomic and demographic correlates predicting a decline in accessing care, and identify the point at which access to care drops. This is critical from a policy perspective, where evidence suggests that while ANC has improved, there is a fall in care during the critical postnatal phase.

Our study provides robust empirical analysis using a large sample. Finally, given the heterogeneity in MCH care observed across the Indonesian provinces, our analysis has significant policy implications in identifying regional gaps in CoC, points at which levels of care utilization drop, and the socioeconomic and demographic factors explaining this fall.

## Methods

### Data

The analysis in this paper is based on four cross-sectional waves of the nationally representative IDHS conducted in the years 2002, 2007, 2012, and 2017. The IDHS is a component of the worldwide Demographic and Health Surveys which is an extensive multi-topic survey that emphasizes MCH. The IDHS is a cross-sectional survey that focuses on women of childbearing age (15–49 years). These women were interviewed using a standard questionnaire across all waves which included detailed questions on the socioeconomic and demographic characteristics of surveyed women and their households, the birth histories of all children born in the five years before the survey, and information relating to the use of healthcare services.

The surveys were implemented by the Indonesian National Family Planning Coordinating Board in conjunction with governmental institutions including the Ministry of Health and Statistics Indonesia. The dataset can be obtained free of cost upon registration from the DHS website.

Our final sample includes 23 574 children aged 12–35 months who were alive at the time of the survey and for whom we have detailed information on their mother’s utilization of health care and the socioeconomic and demographic characteristics of their households. We restrict our analysis to children aged 12–35 months because at 12 months of age, children should have completed their IM schedule. The upper age of the sample is set at 35 months of age because the IDHS 2017 only collects information on childhood IM for children aged up to 35 months.

### Measures

The main outcome measures are the four components of CoC in MCH care. These include the following: (1) at least four ANC visits (ANC4); (2) ANC4 and ID; (3) ANC4, ID, and receipt of PNC within two months of birth; and (4) ANC4, ID, PNC, and child completing full IM. IM is defined as a child receiving all four vaccinations, that is, bacille Calmette-Guérin (BCG), Polio dose 3, diphtheria-pertussis-tetanus (DPT) dose 3, and measles. Note that according to the Indonesian IM schedule, children are given BCG and first dose of Polio at one month, first dose of DPT and the second dose of Polio at 2 months, third dose of Polio at 3 months, and the first dose of Rubella measles at nine months.

The predictor variables in our analysis include an array of demographic and socioeconomic characteristics of the child and their households. These include the mother’s age, maternal and paternal education attainment (no education, primary education, secondary, and above), household size, and number of children in the household aged below five years. The household’s economic status was measured using the wealth index that was available in the IDHS dataset. The wealth index is a composite measure of a household’s cumulative living standards. Using easy-to-collect data on a household’s ownership of selected assets, types of water access, and sanitation facilities, using principal components analysis methods, the households were classified into five quintiles ranging from the poorest to the richest.

Additionally, we also include some measures of the mother’s decision-making autonomy and difficulty in accessing healthcare services. Specifically, the IDHS survey collects information on women’s decision-making autonomy by including self-reported responses to a series of questions such as the following: (1) health care for herself, (2) making major household purchases, and (3) visits to her family or relatives. Based on responses to these questions, the mother is assumed to have no autonomy if she is not involved in decision-making (alone or jointly) in any of the three situations, partially if she is involved in making decisions either alone or with other household members in one or two situations. Finally, if she is involved in decision-making on all three questions, we assume that she has full autonomy. We also include a dummy variable to indicate whether or not the mother has access to (reading newspapers/watching television/listening to radio channels) and another dummy variable (yes, 1, and 0, no) to indicate whether distance to a health facility was a big problem (1 = yes) or not a big problem (0 = no).

### Statistical analysis

We empirically estimated the association between the socioeconomic and demographic characteristics of children and their household and the utilization of CoC. The latent propensity of progression at each stage in the CoC in the four maternal and childcare components for a child 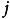 residing in province 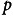 and interviewed in year 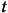 , 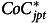 , was assumed to depend on a series of child/mother/household socioeconomic and demographic controls. Unobserved factors 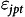 further contribute to the propensity of each stage of the progression in CoC, leading to a latent variable model of the form:
(1)

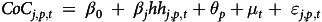




where 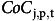 denotes a latent variable, representing one of the four measures of CoC received by child *j* in province *p* at time *t*, and 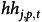 is a vector of control variables and incorporates parental and socioeconomic characteristics of the child’s parents and his/her household. Accordingly, we estimate a series of univariate Probit models for each of the four components of the CoC.

In our estimation, we include indicator variables for provinces *(* 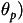 to address potential time-invariant unobserved heterogeneity. We also include an indicator variable for survey years *(* 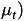 , to control for common changes over time that may affect the status of CoC at the national level.

## Results

**Figure 2.**
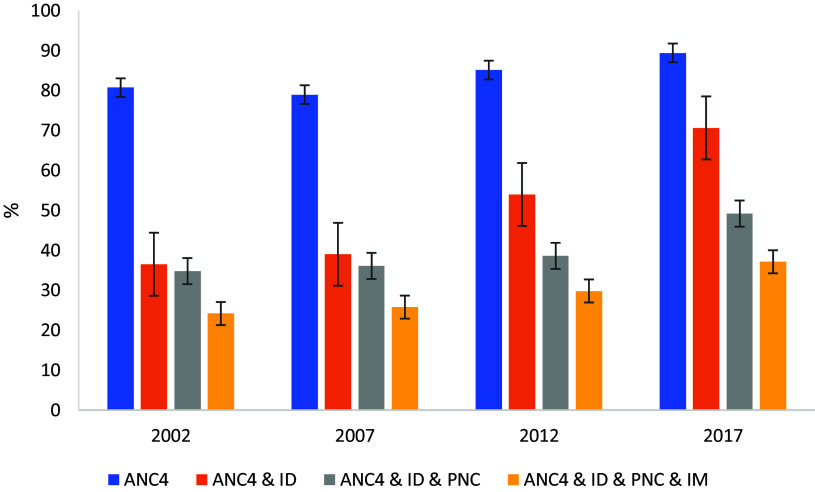
Status of CoC in MCH care during 2002–2017. Source: Authors’ calculation from IDHS from 2002 to 2017. Note: ANC4 = at least four or more antenatal care visits; CoC, continuum of care; ID = institutional delivery; IDHS, Indonesian Demographic and Health Survey; MCH = maternal and child health; PNC = postnatal check; IM = immunization.

**Figure 3.**
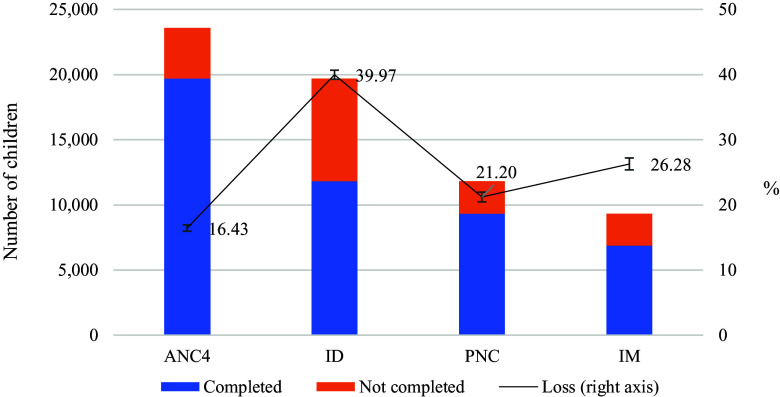
Dropout rate at different stages of CoC in MCH care. Source: Authors’ calculation from IDHS 2002–2017. Note: ANC4 = at least four or more antenatal care visits; CoC, continuum of care; ID = institutional delivery; IDHS, Indonesian Demographic and Health Survey; MCH = maternal and child health; PNC = postnatal check; IM = immunization.

Trends in overall CoC at each stage of care are presented in Figures 2 and 3. Figure 2 shows that across the sample, CoC has improved at each stage over the period 2002–2017. However, only 37.1% of the sample had received all four components of CoC by 2017. Notably, while access to ANC4 remains high, there is a significant decline in care utilization at the postnatal stages. For example, in 2002, while the proportion of the sample that had four or more ANC visits is high (80.7%), only around 36.5% of the samples report having both ANC4 and ID, which drops even further when we include PNC (34.8%). Accordingly, in 2002, <24% of the sample had all four components of CoC. Although there have been some improvements in 2017, the proportion of the sample receiving CoC at the four stages are 89.4% (ANC4), 70.6% (ANC4 and ID), 49.2% (ANC4, ID, and PNC), and less than half the sample (37.1%) receiving the full CoC.

Table 1 presents the summary statistics of the main background characteristics of the sample corresponding to each stage of a child’s CoC. In general, we observe that at each stage of the continuum, those children with better-educated mothers have higher rates of completion of care. For example, 65.6% of children with ANC4 had secondary or higher levels of education (column 1) and 78.5% of children receiving all four components of CoC had mothers with secondary education or higher. Similarly, completion of care at each of the four stages was higher among children with mothers who had better access to media.

**Table 1.** Summary statistics

Background characteristics	Receiving CoC at four stages of MCH care								
	ANC4		ANC4 and ID		ANC4, ID, and PNC		ANC4, ID, PNC, and IM		
	No	Yes	No	Yes	No	Yes	No	Yes	
	1	2	3	4	5	6	7	8	N
Mother’s age									
*15–19 years*	4.9	2.6	4.2	1.9	3.8	1.9	3.6	1.6	734
*20–29 years*	46.6	49.1	50.1	47.7	48.8	48.7	48.9	48.5	11 492
*30–49 years*	48.5	48.3	45.6	50.4	47.5	49.3	47.5	49.9	11 348
Mother’s education									
*No*	9.8	1.5	5.3	0.6	4.4	0.6	3.8	0.4	678
*Primary*	55.9	32.9	52.9	22.8	46.6	23.1	43.5	21.2	8139
*Secondary+*	34.2	65.6	41.8	76.6	49.0	76.3	52.7	78.5	14 757
Father’s education									
*No*	6.4	1.4	3.8	0.7	3.3	0.6	2.9	0.3	523
*Primary*	54.6	32.4	51.1	23.2	45.9	22.8	42.5	21.5	7784
*Secondary+*	38.6	66.0	44.8	76.0	50.5	76.5	54.4	77.9	15 200
Urban	27.4	49.7	27.7	61.5	34.1	61.9	39.5	60.9	10 382
Exposure to mass media	87.6	97.2	92.4	98.7	93.5	98.8	94.4	98.9	22 323
Number of under five children	1.5	1.3	1.4	1.3	1.3	1.3	1.3	1.2	23 574
Mean household size	5.9	5.2	5.4	5.2	5.4	5.2	5.4	5.2	23 574
Distance to health facility: big problem (self-rated)	25.4	11.2	19.5	8.1	17.6	7.7	16.3	6.8	3673
Wealth quintiles									
*Poorest*	47.3	16.9	36.4	9.1	31.6	8.3	27.7	7.8	6793
*Poorer*	22.7	19.6	24.8	16.2	23.4	15.9	22.2	15.7	4737
*Middle*	16.6	20.7	19.7	20.5	19.9	20.5	20.2	20.1	4244
*Richer*	8.3	22.0	13.2	25.6	15.5	25.8	17.5	25.4	3996
*Richest*	5.1	20.8	5.9	28.6	9.5	29.6	12.4	31.0	3804
Mother’s occupation									
*Not working*	51.8	54.1	53.7	53.9	54.3	53.2	54.4	52.6	12 158
*Professional*	1.5	6.0	2.4	7.7	3.4	7.8	3.8	8.5	1436
*Agriculture*	28.3	11.0	23.0	5.8	19.4	6.0	17.3	5.5	3756
*Industry*	6.1	8.3	7.1	8.7	7.3	8.8	7.7	8.6	1461
*Services*	12.3	20.6	13.8	23.9	15.6	24.2	16.8	24.8	4763
Father’s occupation									
*Not working*	9.4	6.5	8.7	5.5	7.6	6.0	7.6	5.5	1592
*Professional*	3.0	8.5	4.3	10.5	5.5	10.6	6.1	11.1	2001
*Agriculture*	47.9	23.6	41.9	15.2	36.7	15.1	33.1	14.5	7414
*Industry*	18.8	25.7	20.7	27.9	23.2	26.6	23.6	26.9	5322
*Services*	21.0	35.7	24.4	41.0	27.0	41.8	29.5	41.9	7245
Mother’s autonomy									
*No*	6.2	4.2	4.9	4.1	5.0	3.8	4.8	3.7	1104
*Partial*	29.0	26.4	28.8	25.0	28.1	25.0	28.2	23.9	5952
*Full*	64.8	69.5	66.3	70.8	66.9	71.2	67.0	72.4	16 518
Year									
*2002*	19.3	80.7	63.5	36.5	65.2	34.8	75.8	24.2	5669
*2007*	21.0	79.0	61.0	39.0	63.9	36.1	74.2	25.8	6049
*2012*	14.9	85.1	46.0	54.0	61.4	38.6	70.2	29.8	5872
*2017*	10.6	89.4	29.4	70.6	50.8	49.2	62.9	37.1	5984

Notes: The table reports percentages (weighted by sample weight) of children by status of continuum of care and background characteristics. *N* refers to the number of women/households that have the corresponding background characteristics, except number of under five children and household size in which *N* refers to sample size. CoC, continuum of care; MCH = maternal and child health; ANC4 = at least four or more antenatal care visits; ID = institutional delivery; PNC = postnatal check; IM = immunization.

Notably, among children receiving all four components of care, only 6.8% of mothers reported that ‘distance to a health facility is a big problem’, whereas it was 16.3% among mothers whose children had not received all four components of care.

Similarly, a higher proportion of urban children and children from the richest wealth quintiles (31%) had completed all four components of care, while only 7.8% of the children from the poorest wealth quintile had completed all four components of care. There is also heterogeneity in access to all four components of care across the continuum, based on parental occupation. Specifically, children with parents working in professional, industrial, or service types of occupation had high CoC completions, relative to children of unemployed or agricultural workers. Finally, we observe higher proportions of children receiving CoC if mothers had higher level of autonomy.

In Figure 3, we further investigate the dropout rate at each stage of the continuum. The dropout rates at the four stages are 16.4%, 40%, 21.2%, and 26.3%, respectively. This suggests that the overall coverage of CoC in the second to the fourth stages is significantly driven by the decline in care at the ID stage, followed by the loss of postnatal care and childhood IM.

**Figure 4.**
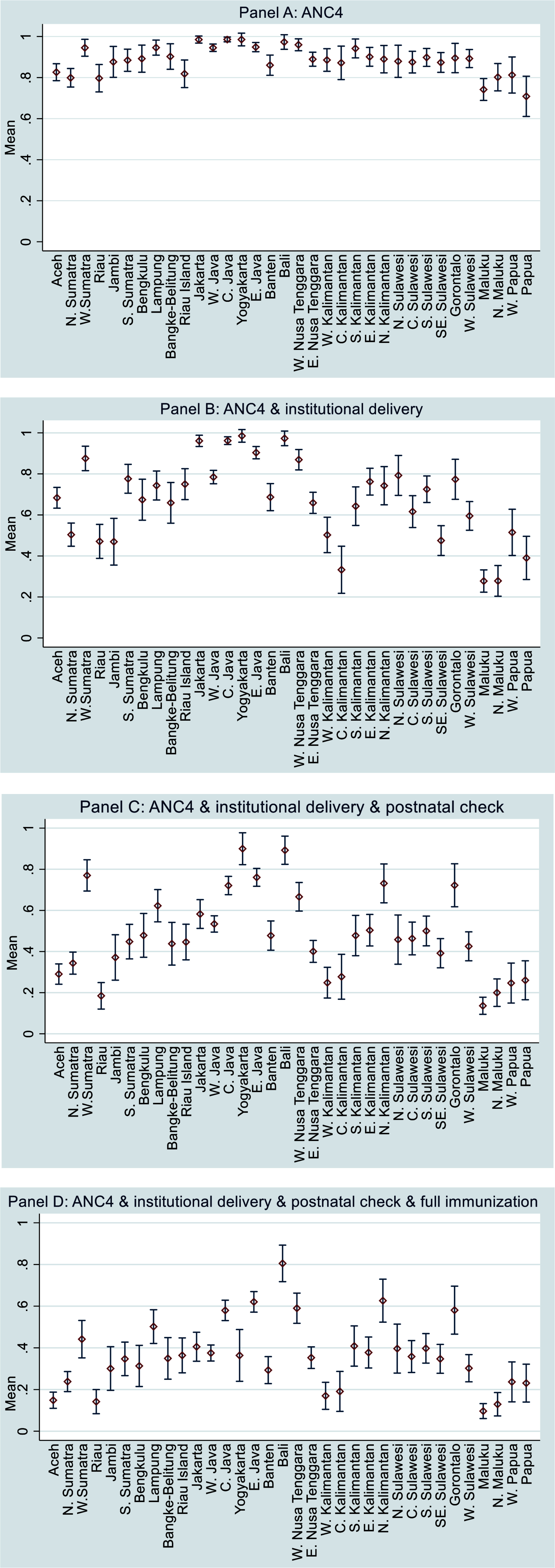
Status of CoC in MCH care by provinces in 2017. Source: Authors’ calculation from IDHS 2017. Figure shows mean value with 95% confidence interval. Note: CoC, continuum of care; IDHS, Indonesian Demographic and Health Survey; MCH = maternal and child health.

In Figure 4, we describe provincial variations in access to each of the components of care. Specifically, while Panel A shows small variations across provinces in accessing ANC4, Panels B, C, and D show large variations in each of the other three components of CoC. We observe lower rates of completion of all four components of care in poorer provinces, such as Aceh (14.9%) or Maluku (9.7%), and higher rates in richer provinces, such as Bali (80.5%), which are consistent with the statistics reported in Table 1.

**Figure 5.**
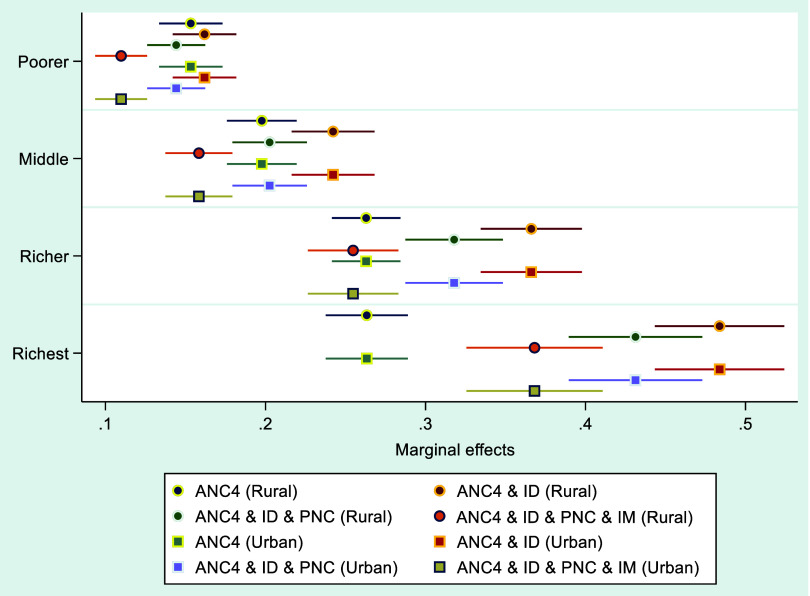
Probit regression estimates: heterogeneous effect of economic status on MCH care at different stages of CoC by place of residence. Source: Authors’ calculation from IDHS 2002 to 2017. Note: ANC4 = at least four or more antenatal care visits; CoC, continuum of care; ID = institutional delivery; IDHS, Indonesian Demographic and Health Survey; MCH = maternal and child health; PNC = postnatal check; IM = immunization. The results control for all other sociodemographic covariates.

### Empirical results

The results of our Probit regression analysis are presented in Table 2 (columns 1–4), with column 1 providing estimates of the probability of receiving ANC4 only, column 2 presenting results for receiving both ANC4 and ID, column 3 presenting results for ANC4, ID, and PNC, and finally column 4 presenting estimates for the completion of all four components of care across the CoC as a consecutive stage of the CoC (i.e., ANC4, ID, PNC, and childhood IM). We report marginal effects.

**Table 2.** Probit estimates

	(1)	(2)	(3)	(4)
	ANC4	ANC4 and ID	ANC4, ID, and PNC	ANC4, ID, PNC, and IM
Mother’s age (ref: 15–19 years)				
*20–29 years*	0.031 ***[0.009, 0.053]	0.033 **[0.003, 0.062]	0.042 **[0.009, 0.075]	0.049 ***[0.015, 0.084]
*30–49 years*	0.035 ***[0.012, 0.057]	0.050 ***[0.020, 0.080]	0.053 ***[0.019, 0.086]	0.059 ***[0.025, 0.093]
Mother’s edu. (ref: no)				
*Primary*	0.083 ***[0.060, 0.106]	0.073 ***[0.036, 0.110]	0.095 ***[0.051, 0.138]	0.096 ***[0.049, 0.144]
*Secondary+*	0.141 ***[0.117, 0.166]	0.155 ***[0.117, 0.194]	0.170 ***[0.125, 0.214]	0.174 ***[0.127, 0.222]
Urban	0.015 **[0.003, 0.028]	0.118 ***[0.103, 0.132]	0.087 ***[0.072, 0.103]	0.051 ***[0.036, 0.065]
Exposure to mass media	0.039 ***[0.021, 0.057]	0.033 **[0.004, 0.061]	0.053 ***[0.021, 0.085]	0.056 ***[0.021, 0.090]
No. of household members (listed)	−0.007 ***[−0.010, −0.005]	−0.006 ***[−0.009, −0.003]	−0.005 ***[−0.008, −0.002]	−0.005 ***[−0.008, −0.002]
No. of children under five	−0.033 ***[−0.041, −0.025]	−0.030 ***[−0.040, −0.020]	−0.032 ***[−0.043, −0.022]	−0.037 ***[−0.047, −0.027]
Dist. to health facility	−0.046 ***[−0.057, −0.034]	−0.034 ***[−0.050, −0.019]	−0.060 ***[−0.077, −0.042]	−0.073 ***[−0.090, −0.055]
Wealth quintiles				
*Poorer*	0.054 ***[0.041, 0.066]	0.066 ***[0.050, 0.081]	0.074 ***[0.056, 0.091]	0.068 ***[0.050, 0.086]
*Middle*	0.073 ***[0.058, 0.087]	0.108 ***[0.091, 0.126]	0.108 ***[0.089, 0.128]	0.092 ***[0.073, 0.112]
*Richer*	0.122 ***[0.105, 0.140]	0.170 ***[0.152, 0.189]	0.154 ***[0.133, 0.175]	0.131 ***[0.111, 0.152]
*Richest*	0.154 ***[0.132, 0.177]	0.267 ***[0.244, 0.289]	0.224 ***[0.201, 0.247]	0.187 ***[0.165, 0.209]
Father’s edu (ref: no)				
*Primary*	0.035 ***[0.010, 0.060]	0.017 [−0.023, 0.057]	0.045 *[−0.002, 0.092]	0.032 [−0.015, 0.079]
*Secondary*	0.057 ***[0.031, 0.083]	0.060 ***[0.020, 0.101]	0.096 ***[0.049, 0.143]	0.079 ***[0.031, 0.126]
Mother’s occupation (ref: not working)				
*Professional*	0.041 ***[0.015, 0.068]	0.021 *[−0.002, 0.044]	0.033 ***[0.010, 0.057]	0.030 ***[0.008, 0.052]
*Agriculture*	−0.014 **[−0.027, −0.001]	−0.036 ***[−0.054, −0.018]	−0.036 ***[−0.056, −0.015]	−0.032 ***[−0.053, −0.011]
*Industry*	0.007 [−0.013, 0.028]	−0.008 [−0.029, 0.014]	0.007 [−0.017, 0.030]	−0.004 [−0.027, 0.018]
*Services*	0.012 *[−0.000, 0.025]	0.007 [−0.006, 0.020]	0.014 *[−0.000, 0.028]	0.004 [−0.009, 0.017]
Father’s occupation (ref: not working)				
*Professional*	0.044 ***[0.018, 0.070]	0.007 [−0.020, 0.034]	0.001 [−0.028, 0.030]	0.013 [−0.014, 0.040]
*Agriculture*	0.005 [−0.014, 0.024]	−0.051 ***[−0.076, −0.027]	−0.052 ***[−0.078, −0.026]	−0.040 ***[−0.064, −0.015]
*Industry*	0.017 *[−0.003, 0.038]	0.000 [−0.024, 0.024]	0.001 [−0.025, 0.026]	0.010 [−0.015, 0.034]
*Services*	0.015 [−0.004, 0.035]	0.002 [−0.020, 0.025]	0.000 [−0.024, 0.025]	0.000 [−0.022, 0.023]
Mother’s autonomy				
*Partial autonomy*	0.015 [−0.007, 0.037]	0.002 [−0.023, 0.027]	0.020 [−0.008, 0.048]	0.013 [−0.014, 0.040]
*Full autonomy*	0.030 ***[0.009, 0.050]	0.020 [−0.004, 0.043]	0.036 ***[0.009, 0.063]	0.033 **[0.007, 0.058]
DHS year (ref: 2002)				
*2007*	−0.008 [−0.024, 0.007]	0.037 ***[0.018, 0.057]	0.029 ***[0.009, 0.050]	0.028 ***[0.010, 0.045]
*2012*	0.024 ***[0.008, 0.039]	0.144 ***[0.124, 0.164]	0.016 [−0.004, 0.036]	0.039 ***[0.021, 0.057]
*2017*	0.049 ***[0.034, 0.065]	0.281 ***[0.260, 0.303]	0.097 ***[0.076, 0.119]	0.093 ***[0.073, 0.112]
Provinces dummies	Yes	Yes	Yes	Yes
Observations	23 574	23 574	23 574	23 574

Notes: The table reports marginal effects; 95% confident intervals are in parenthesis *P < 0.10, **P < 0.05, ***P < 0.01. DHS, Demographic Health Survey; CoC, continuum of care; ANC4 = at least four or more antenatal care visits; ID = institutional delivery; PNC = postnatal check; IM = immunization.

We find that the probability of a child receiving CoC at each of the four stages is significantly associated with maternal age and education and the household’s socioeconomic and demographic characteristics.

Relative to children of young mothers aged 15–19 years, children with mothers aged 20 years and above have a higher probability of accessing services at each of the stages of care and completing the CoC. Specifically, a child whose mother is in the 20–29 years of age category has a 3.1 percentage points higher probability of receiving ANC4 and a 4.9 percentage points higher probability of completing all four components of CoC. Higher coverage of CoC at later ages may be attributed to later marriages, as well as greater personal experience from previous births and pregnancies.

Similarly, a mother’s education is also associated with better CoC outcomes. For example, relative to children of mothers with no education, a child with a secondary educated mother has a 14.1 percentage point higher probability of ANC4 and a higher probably of receiving each of the components of care with a 17.4 percentage points higher probability of completing all four components of the CoC. We see similar positive effects on father’s education, although the size of the marginal effects is smaller than for the mother’s education. Women’s exposure to media (frequently reading newspapers, listening to the radio, and watching TV) is also positively associated with the completion of each stage of the CoC.

Regarding household characteristics, children from urban households have a 1.5–5.1 percentage points higher probability of completing each stage of care from ANC4 to all four components of CoC.

Household wealth status is an important predictor of completion of each stage of CoC, and we observe a positive and monotonic association between household wealth and CoC, with each higher wealth quintile associated with a higher completion of care. Specifically, relative to a child from the poorest wealth quintile, a child would have 13.1 and 18.7 percentage points higher probability of receiving all four components of care if they came from the two highest wealth quintiles, respectively.

Other characteristics that are negatively associated with completing the components of care across the continuum include the number of children under five years of age and household size. These variables likely point to resource constraints that constrain the household’s ability to access the various components of care.

For example, a child with siblings under five years of age has a 3.7 percentage points lower probability of completing all four components of the care. Furthermore, our estimates suggest that a child would be 7.3 percentage points less likely to complete all four components of the continuum if the distance from his/her home to a health facility is a big problem.

Relative to children with non-working mothers, children whose mothers worked in the agricultural sector had a 3.2 percentage points lower probability of completing all four components of care, whereas having mothers in professional occupations increased the probability of completing all four components by three percentage points. For father’s occupations, only agricultural employment is statistically significant, and it is negatively associated with a child completing each of the components of care and all four components of care.

Finally, having mothers with full autonomy is positively associated with a higher probability of care at each of the stages of the continuum relative to a child whose mother has no decision-making autonomy. Specifically, having a mother with full autonomy is associated with a 3, 3.6, and 3.3 percentage points higher probability of having ANC4; ANC4, ID, and PNC; and ANC4, ID, PNC, and IM, respectively.

### Robustness checks

The results in the previous section show that household’s economic status is among the most important predictors for the largest dropout from the CoC in MCH care. To test the consistency of this result, we have conducted some additional robustness checks by estimating Probit models separately for rural and urban residents and by the year of the survey.

The results presented in Figure 5 suggest that irrespective of place of residence, the probability of uptake of care at each stage of the continuum is highest for women in the richest wealth quintile compared with those from the poorest wealth quintile. Furthermore, CoC in MCH care rises monotonically with each higher wealth quintile.

**Figure 6.**
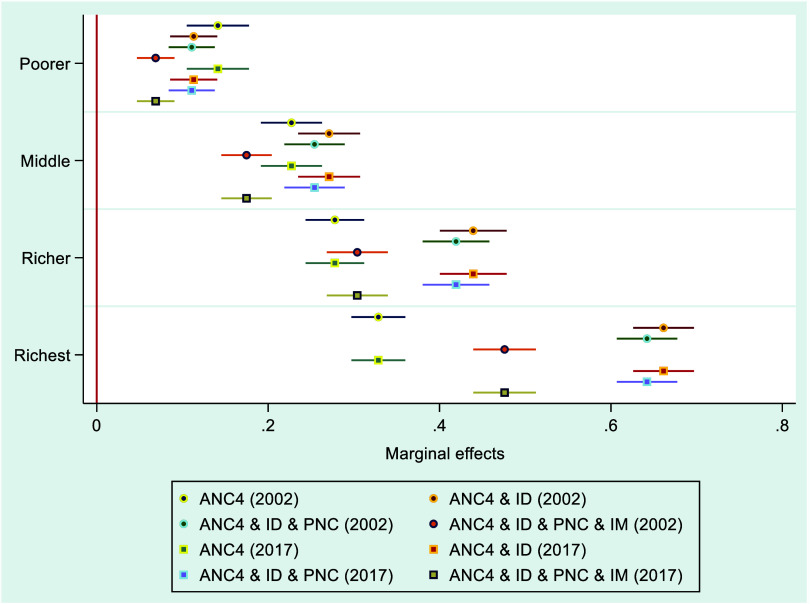
Probit regression estimates: heterogeneous effect of economic status on MCH care at different stages of CoC by year of survey. Source: Authors’ calculation from IDHS 2002 to 2017. Note: ANC4 = at least four or more antenatal care visits; CoC, continuum of care; ID = institutional delivery; IDHS, Indonesian Demographic and Health Survey; MCH = maternal and child health; PNC = postnatal check; IM = immunization. The results control for all other sociodemographic covariates.

Similarly, the results in Figure 6 show that these results are consistent even when we estimate the model separately by survey years.

## Discussion

Our study investigated the level of CoC in MCH care in Indonesia, its coverage at each stage, and its associated socioeconomic and demographic correlates using a large nationally representative sample covering the period 2002–2017 using robust empirical analyses. To the best of our knowledge, ours is the first study from Indonesia that uses four waves of a nationally representative survey to examine the trends in four components of MCH care, using the CoC concept.

We find that although CoC in MCH has improved considerably in Indonesia during the period of the study, <4 in 10 children have received all four components of the CoC. The lowest discontinuation of services occurs between the first and fourth ANC visits, while the highest occurs between ANC4 and ID. Our findings also find evidence of inter-provincial variations in the continuation of MCH services, whereby provinces such as Bali and North and North-East Sulawesi have higher proportions of children completing care across the continuum, whereas the provinces of Aceh, Maluku, and Riau have poorer levels of CoC coverage. Socioeconomic and demographic factors such as household’s economic status, parental (particularly maternal) education, and maternal age are strongly associated with the continuation of CoC at each stage in MCH care. Additionally, proximity to healthcare facilities, urban residence, and maternal autonomy contributed to better coverage and completion of the CoC in MCH care.

Our robustness tests confirm the main findings. Full CoC for the year 2017 is estimated to be 37.1%, while that of those receiving all other services apart from full child IM (ANC4+, ID, and PNC) is above 40%. This is higher than the estimates provided in other studies conducted using just the most recent wave of the IDHS 2017 (Nafiah *et al*., 2022), which could be attributed to the difference in reference period or the components of CoC. Continuous increase in the service utilization for MCH care in Indonesia can also be validated by previous findings indicating a rise in utilization of MCH services (Nababan *et al*., 2017; Andriani *et al*., 2022).

Our findings are in keeping with previous international research. They suggest that although the attainment of four or more ANC constitutes an important component of the CoC, in Indonesia, MCH care services mostly get interrupted in the postpartum and postnatal period. This may be due to poor communication between the mother and the health professionals, lack of skilled birth attendance, and access to emergency obstetric care (WHO, 2005; Kerber *et al*., 2007; Rammohan *et al*., 2021). Furthermore, utilization of PNC and ANC4+ appears to be the most critical components of CoC from an MCH care discontinuation perspective. These results in consistent with previous findings from other developing country contexts. Early initiation of first ANC visits exposes a woman to the processes or channels involved in maternal and childcare, along with information on the type and timing of maternal health services (Wang and Hong, 2015).

Women with secondary or higher levels of education may have better communication skills and knowledge of healthcare services and may potentially be in a position to make informed decisions on pregnancy, maternal, and child healthcare-related services.

Finally, we acknowledge that the study has some limitations. Due to the cross-sectional nature of the dataset, we are unable to draw any causal inferences. Further, the self-reporting nature of the questions in the dataset has the potential to introduce recall bias. Although our study has captured the level of completion of CoC, we are unable to observe the quality of care provided at the health centers. There is also likely to be some geographical variation in the quality of health services and healthcare staff.

## Conclusions

Utilization of MCH services plays a vital role in preventing child and maternal mortality risks. The principle of CoC is an efficient mechanism to improve MCH outcomes by ensuring access to necessary healthcare services and identifying gaps in healthcare utilization (Kerber *et al*., 2007). Our findings show that the CoC continuously declines as women proceed from ANC to other MCH services, with a sharp decline after four ANC visits followed by PNC. Maternal education, urban residence, household’s economic status, and resource constraints are some of the socioeconomic characteristics that affect the utilization of MCH and the possibility of continuing care from one stage to the next over the CoC. Although completion of CoC has the potential of averting antenatal and postnatal complications by seeking timely health care and avoiding delays, improving access to affordable services and quality of care is crucial in maintaining the continuum of MCH services. Access to better education opportunities for female children in particular and provision of better, accessible, and affordable healthcare services in rural areas with better provision for transport facilities have the potential to improve MCH outcomes. Targeted resources and strengthening of current healthcare facilities and related infrastructure in poorer performing provinces are critical for improving MCH care and reducing the gaps in health outcomes between the richer and the poorer provinces.

From a policy perspective, our study has identified the gaps in healthcare utilization at various phases of the CoC and key socioeconomic characteristics of women and children that increase their probability of failing to access care. These can be addressed to improve access to care across the continuum. Further, a better understanding of inter-provincial variations in CoC and greater resources for the poorer performing provinces can help in improving MCH outcomes in Indonesia.

## Data Availability

The dataset is publicly available and can be obtained free of cost upon registration from the following website: https://dhsprogram.com/.

## Declarations

All methods were performed in accordance with the relevant guidelines and regulations.
